# Draft Genome Sequence of *Bacillus* sp. Strain IGA-FME-2, Isolated from the Bulk Soil of Soybean (Glycine max L.) in Northeast China

**DOI:** 10.1128/MRA.00004-21

**Published:** 2021-04-22

**Authors:** Zhenhua Yu, Sergio de los Santos-Villalobos, Yansheng Li, Jian Jin, Fannie Isela Parra Cota, Valeria Valenzuela Ruiz, Guanghua Wang

**Affiliations:** a Key Laboratory of Mollisols Agroecology, Northeast Institute of Geography and Agroecology, Chinese Academy of Sciences, Harbin, China; b Instituto Tecnológico de Sonora, Ciudad Obregón, Sonora, Mexico; c Campo Experimental Norman E. Borlaug, Instituto Nacional de Investigaciones Forestales, Agrícolas y Pecuarias, Ciudad Obregón, Sonora, Mexico; University of Delaware

## Abstract

Here, we present the draft genome of *Bacillus* sp. strain IGA-FME-2. This strain was isolated from the bulk soil of soybean (Glycine max L.). Its genome consists of 3,810 protein-coding genes, 44 tRNAs, two 16S rRNAs, and a single copy of 23S rRNA, with a GC content of 46.4%.

## ANNOUNCEMENT

Soybean (Glycine max L.) is cultivated worldwide due to its nutritional value and oil-yielding characteristics (1). Successful soybean production is hampered by abiotic and biotic stresses such as drought, weeds, insect pests, and diseases, which reduce yields by up to 40% and increase the cost of cultivation (2, 3). Thus, sustainable agricultural practices, such as the application of plant growth-promoting bacteria (4–8), are needed to combat these stresses.

**FIG 1:**
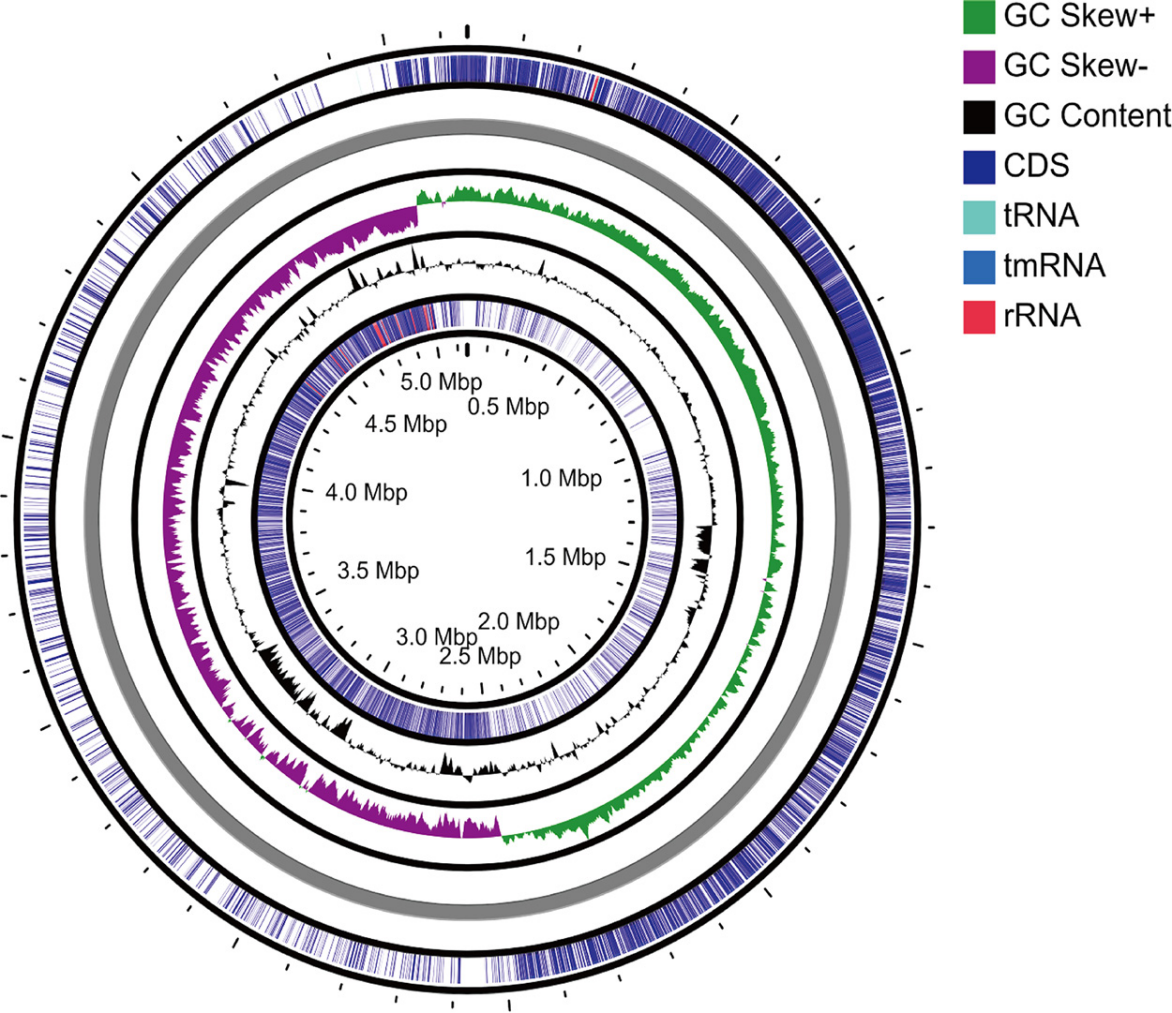
Circular chromosome map of *Bacillus* sp. strain IGA-FME-2, showing the distribution of coding DNA sequences (CDS), tRNAs, rRNAs, and GC content skew (50% of the total base pair window).

Strain IGA-FME-2 was isolated from the bulk soil of soybean in an agricultural field in Harbin, Heilongjiang, China (45°41′N, 126°38′E). One gram of collected soil was suspended in 9 ml of sterile (treated at 121°C and 15 lb/in^2^ for 15 min) distilled water and homogenized for 15 min at 5 × *g* (using a rotary shaker). The soil suspension was serially diluted from 10^−1^ to 10^−6^, 0.1 ml of each dilution was pipetted out and spread onto plates with solid LB medium, and the plates were incubated at 28°C for 48 h. Bacterial colonies were purified by sequential streaking onto solid LB medium, and macroscopic/microscopic characterization was carried out (in triplicate) to ensure axenicity. All isolates obtained were cryopreserved at −80°C using liquid LB medium and 30% glycerol. Then, high-quality genomic DNA was extracted and purified from strain IGA-FME-2, after growth in liquid LB medium at 28°C for 48 h, by using the E.Z.N.A. bacterial DNA kit (Omega, USA) according to the manufacturer’s instructions. The purified DNA was quantified with a TBS-380 fluorometer (Turner BioSystems, Inc., Sunnyvale, CA). High-quality DNA (optical density at 260 nm [OD_260_]/OD_280_, 1.8 to 2.0; total amount of DNA, ≥1 μg; concentration, ≥20 ng/μl) was used for sequencing with the HiSeq 2000 (2 × 100 bp) platform (Illumina, USA). Next-generation sequencing library preparation was carried out by using the NEXTflex rapid DNA-Seq kit version 2.0 for Illumina platforms, according to the manufacturer’s instructions. The quality of the raw reads obtained was analyzed by FastQC version 0.11.5 (9), and all parameters showed very good quality except for sequence duplication levels and per-base sequence content, which showed intermediate quality. Trimmomatic version 0.32 was used to remove adapter sequences by using the universal adapter sequences, low-quality bases by using a sliding window approach of 4:24, and bases at the start of the read, according to the sequencing quality, by using HEADCROP. With these parameters, only 1.95% of reads were dropped, and after a FastQC reanalysis, all parameters were significantly improved to very good quality. Subsequently, a *de novo* assembly was generated by SPAdes version 3.14.1 (10), using the parameter –careful for error correction in reads and –cov-cutoff auto. The draft genome of strain IGA-FME-2 consisted of 40 contigs (>200 bp) (minimum, 209 bp; maximum, 984,802 bp; *N*_50_, 436,298 bp; *L*_50_, 3). The final assembly contained 3,955,933 bp, with a GC content of 46.4%. The assembled contigs were ordered by Mauve Contig Mover version 2.4.0 (11), using the reference genome of Bacillus velezensis FZB42 (GenBank accession number GCA_000015785.1). The reference genome was selected based on the greatest 16S rRNA identity (100%). The circular chromosome map was then generated using the CGView Server (12) (Fig. 1).

Genome annotation was performed by Prokka v 1.11.0 (13). The genome is predicted to contain 3,810 protein-coding genes, 44 tRNAs, 2 16S rRNAs, and a single copy of 23S rRNA.

### Data availability.

This draft genome sequence has been deposited in DDBJ/ENA/GenBank under accession number JADDHM000000000. The version described in this paper is the first version, JADDHM010000000, under BioProject number PRJNA668193 and BioSample number SAMN16400875. Raw data have been deposited in the NCBI SRA under accession number PRJNA675398 (sample IGA-FME-2 [BioSample number SAMN16706821 and SRA accession number SRX9460425]).
